# Anti-PTK7 Monoclonal Antibodies Exhibit Anti-Tumor Activity at the Cellular Level and in Mouse Xenograft Models of Esophageal Squamous Cell Carcinoma

**DOI:** 10.3390/ijms232012195

**Published:** 2022-10-13

**Authors:** Jae Hoon Kim, Won-Sik Shin, Se-Ra Lee, Sanggil Kim, So-Young Choi, Seung-Taek Lee

**Affiliations:** 1 Department of Biochemistry, College of Life Science and Biotechnology, Yonsei University, Seoul 03722, Korea; 2 New Drug Development Center, Osong Medical Innovation Foundation, Cheongju 28160, Chungbuk, Korea

**Keywords:** protein tyrosine kinase 7 (PTK7), monoclonal antibody, esophageal squamous cell carcinoma, tumorigenesis, anticancer agent

## Abstract

PTK7 is a catalytically defective receptor protein tyrosine kinase upregulated in various cancers, including esophageal squamous cell carcinoma (ESCC). In previous studies, we observed a positive correlation between PTK7 expression levels and tumorigenicity in various ESCC cell lines and xenograft mice with ESCC KYSE-30 cells. In this study, we analyzed the effects of anti-PTK7 monoclonal antibodies (mAbs) on the tumorigenic activity in KYSE-30 cells and in mouse xenograft models. PTK7 mAb-32 and mAb-43 bind with a high affinity to the extracellular domain of PTK7. PTK7 mAbs significantly reduced three-dimensional cell proliferation, adhesion, wound healing, and migration. PTK7 mAbs also reduce chemotactic invasiveness by decreasing MMP-9 secretion. PTK7 mAbs decreased actin cytoskeleton levels in the cortical region of KYSE-30 cells. PTK7 mAbs reduced the phosphorylation of ERK, SRC, and FAK. In a mouse xenograft model of ESCC using KYSE-30 cells, PTK7 mAbs reduced tumor growth in terms of volume, weight, and the number of Ki-67-positive cells. These results demonstrated that PTK7 mAbs can inhibit the tumorigenicity of ESCC at the cellular level and in vivo by blocking the function of PTK7. Considering the anticancer activities of PTK7 mAbs, we propose that PTK7 mAbs can be used in an effective treatment strategy for PTK7-positive malignancies, such as ESCC.

## 1. Introduction

Protein tyrosine kinase 7 (PTK7) is a catalytically defective receptor protein tyrosine kinase (RPTK) whose catalytic function is inactivated by alterations in the tyrosine kinase domain. Despite the lack of catalytic activity, catalytically defective RPTKs play an important role in the regulation of cell signaling through interactions with other proteins, such as catalytically active RPTKs [1,2]. Therefore, some catalytically defective RPTKs involved in tumorigenesis, such as PTK7, Erb-B2 receptor tyrosine kinase 3 (ErbB3), and EPH receptor A10 (EphA10), are considered target molecules for cancer therapy [3,4,5,6,7].

The expression of PTK7 is upregulated and is associated with tumorigenicity in diverse cancers, including esophageal squamous cell carcinoma (ESCC) and breast cancer [3,8,9]. For example, patients with high PTK7 expression had lower survival rates than those with low PTK7 expression in ESCC and triple-negative breast cancer (TNBC) [3,10]. In addition, PTK7 expression is correlated with higher cell proliferation, survival, migration, and invasion in ESCC cells [3,11] and breast cancer cells [8], as well as enhanced tumor growth in mouse xenograft models using ESCC and TNBC cells [12,13].

Several studies have been conducted to treat cancers by targeting PTK7 or to predict the prognosis of tumors based on the degree of PTK7 expression. A PTK7-targeted antibody–drug conjugate (ADC) induced regression of PTK7-positive tumors [4]. PTK7 aptamers conjugated with a detection probe or chemical have shown potential for use as molecular imaging agents or therapeutic agents in PTK7-positive tumors [5,14]. Recently, a small-molecule inhibitor that interferes with the interaction between PTK7 and β-catenin exhibited anticancer effects in colorectal cancer by blocking the Wnt/β-catenin pathway [15].

Esophageal cancer (EC) ranks seventh in new cancer incidence and sixth in cancer-related deaths worldwide. EC can be classified into two histological subtypes with different etiologies: ESCC and esophageal adenocarcinoma (EAC) [16]. ESCC is mainly found in East Asia and sub-Saharan Africa, and its incidence is associated with excessive drinking, smoking, and intake of hot food and drinks. In contrast, EAC is mainly found in high-income Western countries, and its incidence is associated with overweight and gastroesophageal reflux.

Most ESCCs rarely exhibit symptoms during the early stages of progression. Therefore, the patient’s mortality rate is high, and the prognosis is poor [17]. Currently, surgery, radiotherapy, and chemotherapy with 5-FU, cisplatin, adriamycin, and paclitaxel are the main treatments. For the targeted treatment of ESCC, only a small number of options are available, including targeting epidermal growth factor receptor (EGFR), human epidermal growth factor receptor 2 (HER2), or vascular endothelial growth factor (VEGF) using a targeted inhibitor or neutralizing antibody and a combination of targeted therapy and chemotherapy. However, the treatment efficacy is limited owing to the acquisition of resistance to tumor cells by targeted therapy and the uncertainty of the synergistic effects of the combination therapy [18,19]. Therefore, the current treatment still has a high recurrence rate and low treatment success rate, leaving an unmet need for a new treatment.

We previously showed that PTK7 knockdown reduced the proliferation, wound healing, and migration of ESCC cells, such as TE-10 [3]. In addition, tumor growth was reduced by PTK7 knockdown in a xenograft model of ESCC using KYSE-30 cells [12]. However, there is a limitation in developing small-molecule inhibitors to reduce tumorigenesis of ESCC because PTK7 is catalytically inactive, and the molecular mechanism of PTK7 has not yet been elucidated in ESCC. Therefore, we generated monoclonal antibodies (mAbs) against PTK7 and measured the binding affinity of PTK7 mAbs to soluble PTK7 (sPTK7). We then analyzed whether PTK7 mAbs could inhibit oncogenic phenotypes such as cell proliferation, adhesion, wound healing, migration, and invasion and whether they affect the organization of actin cytoskeleton in KYSE-30 cells. In addition, we analyzed whether PTK7 mAbs could reduce the activation of oncogenic signaling proteins in KYSE-30 cells. Finally, we examined the effect of PTK7 mAbs on tumorigenesis in mouse xenograft models of ESCC. From this study, we evaluated the potential of PTK7 mAbs as anticancer agents for the treatment of PTK7-positive cancers, including ESCC.

## 2. Results

### 2.1. PTK7 mAbs Bind to sPTK7 with High Affinity

To develop PTK7 mAbs that block PTK7 function, we generated mouse mAbs against human sPTK7 as an antigen using hybridoma technology. Two clones, PTK7 mAb-32 and 43, which bind specifically to sPTK7, were chosen for further study.

**Figure 1:**
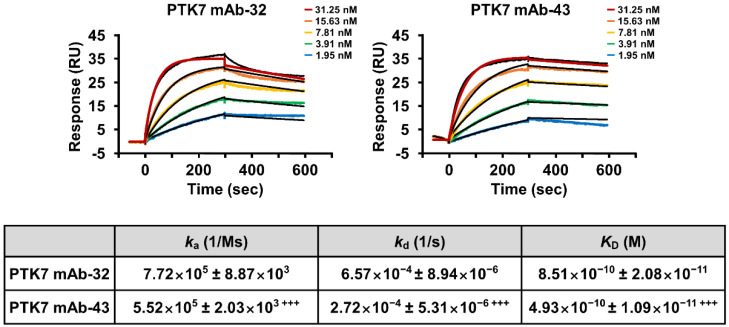
PTK7 mAbs bind to sPTK7 with high affinity. The binding affinities of PTK7 mAb-32 and PTK7 mAb-43 were measured using SPR analysis. The His-tagged sPTK7 polypeptide was immobilized on a sensor chip as a ligand and increasing concentrations of PTK7 mAbs were injected into the sensor chip chamber. The measured kinetic constants are presented in a table below the sensograms. Each value in the inserted table represents the mean ± SD of three independent experiments: +++ *p* < 0.001 vs. PTK7 mAb-32.

The binding affinity of PTK7 mAbs to sPTK7 was measured by surface plasmon resonance (SPR) analysis by flowing PTK7 mAbs as analytes over His-tagged sPTK7 immobilized on the His-capture chip. Both antibodies showed excellent binding affinity to sPTK7, but PTK7 mAb-43 (*K*_D_ = 4.93×10^−10^ ± 1.09×10^−11^ M) showed significantly stronger binding than PTK7 mAb-32 (*K*_D_ = 8.51×10^−10^ ± 2.08×10^−11^ M) (Figure 1).

### 2.2. PTK7 mAbs Reduce Cell Proliferation, Adhesion, Wound Healing, and Migration in ESCC KYSE-30 Cells

**Figure 2:**
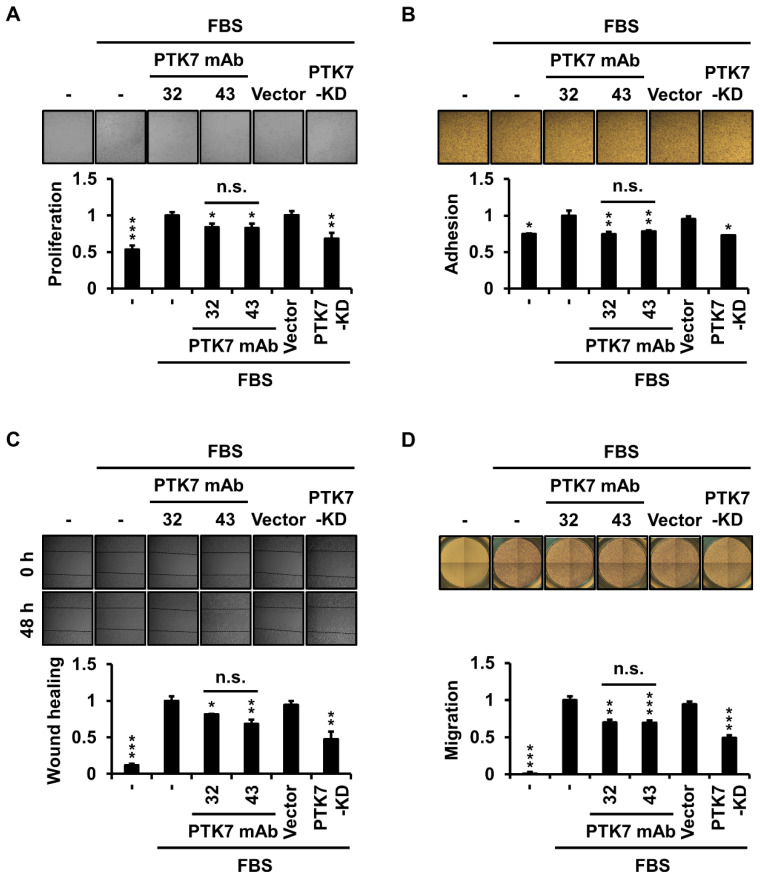
PTK7 mAbs reduce 3D cell proliferation, adhesion, wound healing, and migration in KYSE-30 cells. KYSE-30 cells were incubated in Dulbecco’s modified Eagle medium/nutrient mixture F-12 (DMEM/F12) medium with or without 2% fetal bovine serum (FBS) in the presence of PTK7 mAb (10 μg/mL) for the indicated time intervals. KYSE-30 cells infected with the vector or PTK7 knockdown viruses were used as controls. (**A**) Cell proliferation was analyzed after incubation for 72 h in a 3D culture system in which the cells were embedded in Matrigel. Viable cells were measured using the WST-8 assay. Representative micrograph images (×100) taken at 72 h after plating are shown. (**B**) Cell adhesion was analyzed 1 h after plating in a 96-well plate pre-coated with type I collagen. The adherent cells were stained with crystal violet. Representative micrographs (×100) are shown. (**C**) Wound healing was analyzed 48 h after wounding in a monolayer of cells. Representative micrograph images (×100) taken at 0 and 48 h after wounding are shown. (**D**) Chemotactic migration was analyzed after incubation for 24 h in a Transwell plate. The migrated cells were stained with crystal violet. Representative micrographs (×40) are shown. The quantitation of each assay is described in the Materials and Methods section. Each bar represents the mean ± SD of three independent experiments: * *p* < 0.05, ** *p* < 0.01, or *** *p* < 0.001 vs. cells treated with FBS alone; n.s. (not significant) between PTK7 mAb-32 and PTK7 mAb-43.

To determine whether PTK7 mAbs affected ESCC cell proliferation, we assessed cell proliferation by Matrigel-embedded three-dimensional (3D) culture of KYSE-30 cells for 3 days. Serum depletion and PTK7 knockdown groups showed a significant decrease in proliferation to 53.5 ± 5.2% and 68.5 ± 7.5%, respectively, compared with that of the serum-treated control group. Treatment with PTK7 mAb-32 and -43 significantly reduced 3D cell proliferation to 84.1 ± 4.9% and 83.1 ± 6.2%, respectively (Figure 2A). To analyze the effect of PTK7 mAbs on the adhesion of ESCC cells, we analyzed the change in the adhesion of KYSE-30 cells to type 1 collagen, the most abundant component of the extracellular matrix. Serum depletion and PTK7 knockdown groups showed a significant reduction in adhesion to 74.8 ± 1.1% and 73.0 ± 0.2%, respectively, compared with that of the serum-treated control group. Treatment with PTK7 mAb-32 and -43 significantly reduced adhesion by 74.8 ± 2.8% and 78.6 ± 1.4%, respectively (Figure 2B). We then analyzed whether wound healing and chemotactic migration of ESCC cells were altered by the PTK7 mAb treatment. In the wound healing assay, when PTK7 knockdown in KYSE-30 cells showed a significant reduction to 47.6 ± 10.3% compared with that of the serum-treated control group, treatment with PTK7 mAb-32 and -43 in KYSE-30 cells significantly reduced to 81.5 ± 0.6% and 68.6 ± 5.6%, respectively (Figure 2C). In the chemotactic migration assay, when PTK7 knockdown showed a significant reduction to 49.1 ± 3.5%, compared with that of the serum-treated control group, treatment of PTK7 mAb-32 and -43 in KYSE-30 cells significantly reduced to 70.1 ± 3.6% and 69.6 ± 3.1%, respectively (Figure 2D). These results demonstrate that the selected PTK7 mAbs exert an anti-tumor effect at the cellular level by interfering with the oncogenic function of PTK7 in KYSE-30 cells.

### 2.3. PTK7 mAbs Reduce Invasiveness and Matrix Metalloproteinase (MMP)-9 Secretion in ESCC KYSE-30 Cells

**Figure 3:**
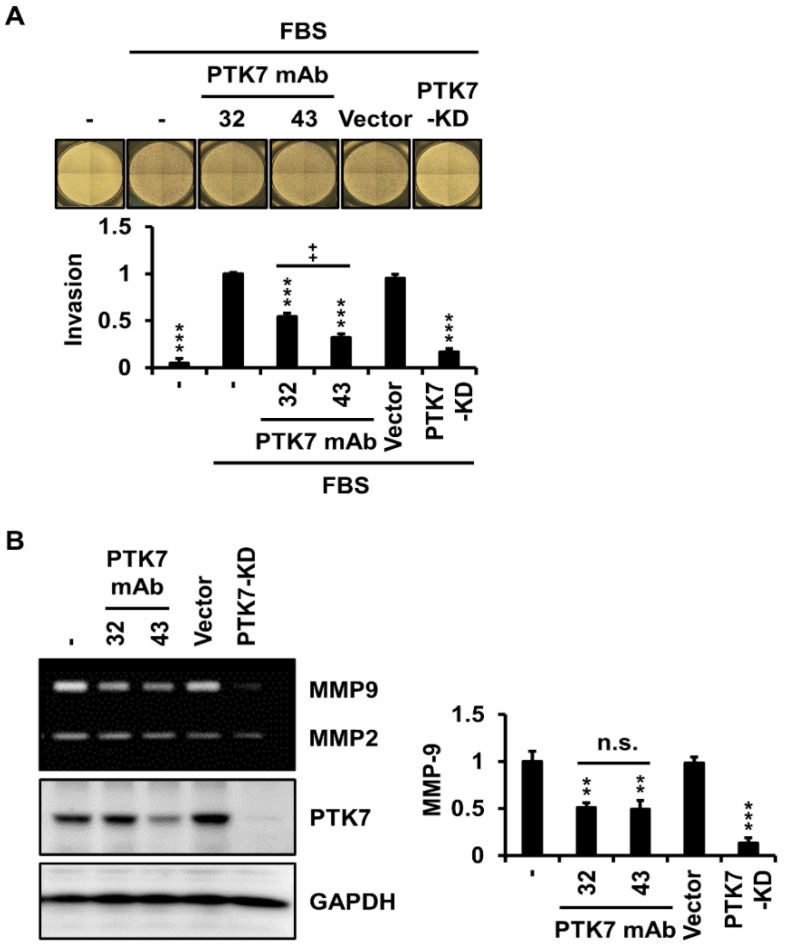
PTK7 mAbs reduce cell invasion and MMP-9 secretion in KYSE-30 cells. KYSE-30 cells infected with the vector or PTK7 knockdown viruses were used as controls. (**A**) Chemotactic invasion was analyzed after incubation of KYSE-30 cells in DMEM/F12 medium with or without 2% FBS in the presence of PTK7 mAb (10 μg/mL) for 48 h using a Transwell plate. The invaded cells were stained with crystal violet. Representative micrographs (x40) are shown. Quantification of the invasion assay is described in the Materials and Methods section. Each bar represents the mean ± SD of three independent experiments: *** *p* < 0.001 vs. cells treated with FBS alone; ++ *p* < 0.01 between PTK7 mAb-32 and PTK7 mAb-43. (**B**) MMP-2 and MMP-9 levels in the conditioned medium of KYSE-30 cells, which were incubated in serum-free DMEM/F12 medium with or without PTK7 mAbs (10 μg/mL) in the presence of TNF-α (1 ng/mL) for 24 h, were analyzed using gelatin zymography. Protein levels in zymograms were quantitated using ImageJ software (version 1.52). Each bar represents the mean ± SD of three independent experiments: ** *p* < 0.01 or *** *p* < 0.001 vs. cells treated with TNF-α alone; n.s. (not significant) between PTK7 mAb-32 and PTK7 mAb-43.

To examine whether PTK7 mAbs affect the invasion of ESCC cells, we analyzed chemotactic invasion and the expression of gelatinolytic MMPs, MMP-2 and MMP-9, using gelatin zymography in KYSE-30 cells with or without PTK7 mAb treatment. When PTK7 knockdown showed a significant reduction in invasion to 16.8 ± 3.7% compared with that of the serum-treated control group, treatment with PTK7 mAb-32 and -43 significantly reduced invasion to 54.5 ± 3.7% and 32.1 ± 4.1%, respectively (Figure 3A). Since MMP-2 and MMP-9 levels secreted from KYSE-30 cells were low, it was difficult to detect changes in MMP levels using gelatin zymography following treatment with PTK7 mAbs (Supplementary Figure S1). In the presence of 1 ng/mL tumor necrosis factor-α (TNF-α), MMP-9 secretion was clearly detectable using gelatin zymography. Under this condition, MMP-9 secretion was significantly reduced by treatment of PTK7 mAb-32 (51.1 ± 4.8%) and -43 (49.2 ± 9.4%) as well as PTK7 knockdown (13.2 ± 5.4%), compared with that of the control group (Figure 3B). Treatment with TNF-α (1 ng/mL) in KYSE-30 cells did not increase the secreted MMP-2 level, so the effect of PTK7 mAbs on MMP-2 secretion could not be unequivocally assessed. These results demonstrated that PTK7 mAb reduced the invasiveness of KYSE-30 cells by inhibiting MMP-9 secretion.

### 2.4. PTK7 mAbs Inhibit Actin Polymerization in the Cortical Area of KYSE-30 Cells

**Figure 4:**
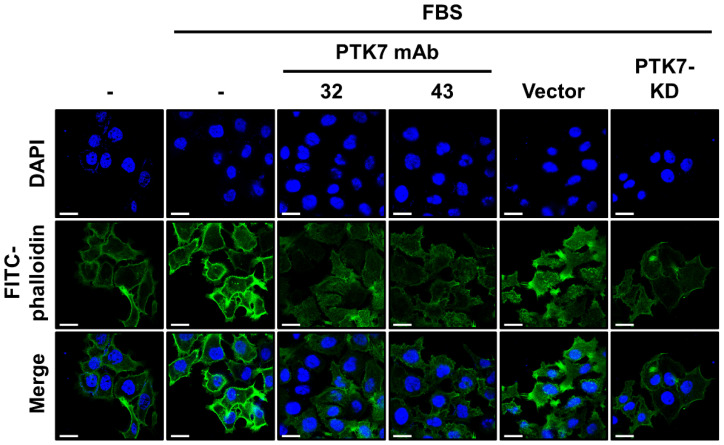
PTK7 mAbs inhibit actin polymerization in KYSE-30 cells. KYSE-30 cells were incubated in DMEM/F12 medium with or without 2% FBS in the presence of PTK7 mAb (10 μg/mL) for 24 h. KYSE-30 cells infected with the vector or PTK7 knockdown viruses were used as controls. The cells were fixed with 3.7% paraformaldehyde (PFA), and the actin filaments (green) and nuclei (blue) were stained with FITC-conjugated phalloidin and 4′,6-diamidino-2-phenylindole (DAPI), respectively. The scale bar represents 20 μm.

As PTK7 mAbs were able to significantly impair the migration and invasion of KYSE-30 cells, we analyzed whether PTK7 mAbs could affect actin cytoskeleton remodeling, which is essential for migration and invasion. Actin filaments were detected using fluorescence staining with fluorescein isothiocyanate (FITC)-conjugated phalloidin. In the serum-treated control groups (FBS/- and FBS/vector), strong polymerization of actin filaments was observed in the cortical area of KYSE-30 cells, particularly at the leading edges and focal contacts. In contrast, serum depletion and PTK7 knockdown significantly reduced actin polymerization. Treatment with PTK7 mAb-32 and -43 reduced actin polymerization to a level similar to that observed with PTK7 knockdown (Figure 4).

### 2.5. PTK7 mAbs Not Only Inhibit Activation of Oncogenic Signaling Proteins but Also Reduce PTK7 Levels in KYSE-30 Cells

**Figure 5:**
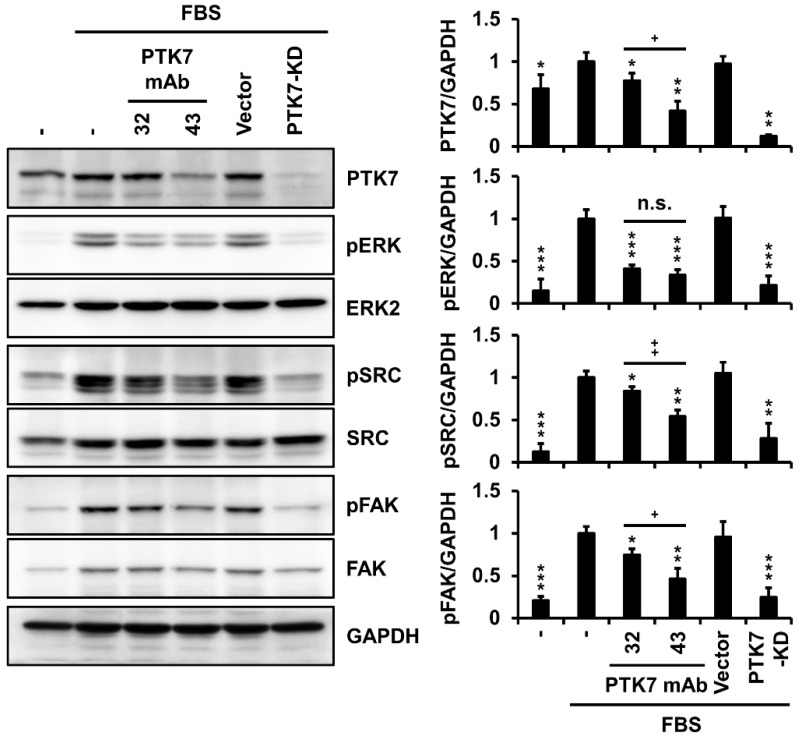
PTK mAbs inhibit activation of ERK, SRC, and FAK in KYSE-30 cells. KYSE-30 cells were incubated in DMEM/F12 medium with or without 2% FBS in the presence of PTK7 mAb (10 μg/mL) for 24 h. KYSE-30 cells infected with the vector or PTK7 knockdown viruses were used as controls. Cell lysates were subjected to Western blot analysis using PTK7, pERK, ERK, pSRC, SRC, pFAK, FAK, and GAPDH antibodies. Phosphoprotein/GAPDH ratio was quantitated using ImageJ software. Each bar represents the mean ± SD of three independent experiments: * *p* < 0.05, ** *p* < 0.01, or *** *p* < 0.001 vs. cells treated with FBS alone; + *p* < 0.05, ++ *p* < 0.01, or n.s. (not significant) between PTK7 mAb-32 and PTK7 mAb-43.

Signaling proteins, such as extracellular signal-regulated kinase (ERK), SRC, and focal adhesion kinase (FAK), are involved in pathways that control oncogenic phenotypes, including cell proliferation, adhesion, wound healing, migration, and invasion of ESCC cells [3,11,12]. We investigated whether treatment with PTK7 mAbs could alter the activation of these signaling proteins in KYSE-30 cells. Compared with serum depletion, the presence of serum increased the phosphorylation of ERK, SRC, and FAK. When PTK7 knockdown showed a significant reduction in phosphorylation of ERK (12.3 ± 1.6%), SRC (21.5 ± 10.9%), and FAK (24.6 ± 11.1%) compared with that of the serum-treated control group, treatment with PTK7 mAbs significantly reduced phosphorylation of ERK (40.9 ± 3.7% for mAb-32 and 33.7 ± 6.0% for mAb-43), SRC (84.0 ± 4.9% for mAb-32 and 54.3 ± 7.1% for mAb-43), and FAK (74.7 ± 6.9% for mAb-32 and 46.5 ± 12.1% for mAb-43), respectively (Figure 5). Interestingly, PTK7 mAbs treatment significantly reduced the expression of PTK7 in KYSE-30 cells. Treatment with PTK7 mAb-43 (42.1 ± 11.2%) was significantly more potent in reducing PTK7 expression levels in KYSE-30 cells than treatment with PTK7 mAb-32 (77.3 ± 8.9%) (Figure 5), suggesting antibody-mediated endocytosis and lysosomal degradation of PTK7.

### 2.6. PTK7 mAbs Reduce Tumor Volume, Weight, and Number of Proliferating Cells in a Xenograft Mouse Model of ESCC

**Figure 6:**
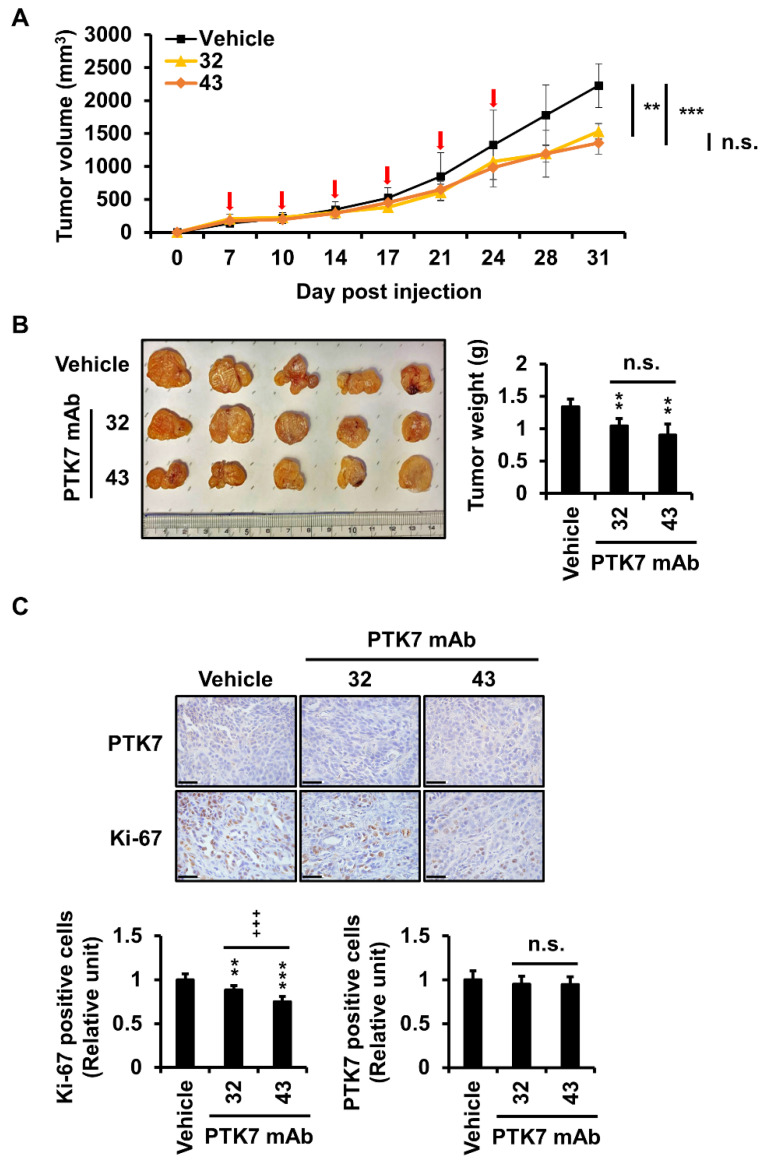
PTK7 mAbs inhibit tumor growth in an ESCC xenograft mouse model. A xenograft mouse model of ESCC was established by the subcutaneous injection of KYSE-30 cells into the dorsal regions of nude mice. When cell masses were recognized after cell injection, PTK7 mAb (10 mg/kg) was injected intraperitoneally twice a week for three weeks. The mice were sacrificed 7 days after PTK7 mAb injection and the tumors were excised. (**A**) Tumor volume was measured in the xenograft mice. (**B**) Images of tumors excised from sacrificed mice. Tumor weight is shown on a graph. Each bar represents the mean ± SD of five mice. (**C**) IHC staining of PTK7 and Ki-67. Representative images are shown. The scale bar represents 50 μm. Protein levels in IHC images were quantitated using ImageJ software. Each bar represents the mean ± SD of five mice: ** *p* < 0.01 or *** *p* < 0.001 vs. vehicle; +++ *p* < 0.001 or n.s. (not significant) between PTK7 mAb-32 and PTK7 mAb-43.

Since KYSE-30 cells can generate tumors in a xenograft mouse model, the anticancer effect of PTK7 mAbs was analyzed in a xenograft mouse model of ESCC using KYSE-30 cells. When tumor formation was observed after subcutaneous injection of KYSE-30 cells into the back, PTK7 mAbs (10 mg/kg) were injected intraperitoneally twice a week for three weeks. After injection of PTK7 mAbs, inflammation was detected at the tumor sites in some mice, particularly in the vehicle group. Therefore, xenograft mice were sacrificed one week after the last antibody injection, which was one week earlier than originally planned. Nevertheless, tumor volumes were significantly reduced to 68.8 ± 5.3% by PTK7 mAb-32 and to 61 ± 7.7% by PTK7 mAb-43, compared with that of the vehicle group (Figure 6A). Tumor weights were also significantly reduced to 77.6 ± 8.5% by PTK7 mAb-32 and to 67.2 ± 12.9% by PTK7 mAb-43, compared with that of the vehicle group (Figure 6B). Although PTK7 mAb-43 had lower mean values than did PTK7 mAb-32 for tumor volume and weight, there was no statistically significant difference between PTK7 mAb-43 and PTK7 mAb-32.

Immunohistochemical (IHC) staining for PTK7 and Ki-67 proteins was performed on tumor sections. Treatment with PTK7 mAb-32 and -43 significantly reduced levels of the Ki-67 protein, a marker for proliferating cells, to 88.7 ± 4.2% and 73.8 ± 4.5%, respectively, compared with that in the vehicle group, in xenograft tumors (Figure 6C). PTK7 mAb-43 showed a statistically significant reduction in Ki-67 staining level compared to PTK7 mAb-32. However, there was no significant difference in PTK7 levels between the vehicle group and the PTK7 mAb-treated groups or between the PTK7 mAb-32 and PTK7 mAb-43 groups.

## 3. Discussion

PTK7 upregulation is inversely proportional to the overall survival rate of patients with ESCC [3]. Additionally, PTK7 knockdown in ESCC cells reduced oncogenic phenotypes and decreased FAK, AKT, and ERK signaling [12]. PTK7 overexpression in prostate cancer patients can predict lymph node metastasis, poor overall survival, and recurrence-free survival [20]. PTK7 expression in colorectal cancer and colorectal adenoma is significantly higher than that in non-tumor mucosa and is significantly correlated with tumor differentiation, lymph node metastasis, and poor survival [21]. Therefore, PTK7 is a biomarker and a therapeutic target molecule for various cancers, including ESCC.

Since PTK7 is a catalytically defective RPTK, in which the catalytic function is inactivated due to alteration of the tyrosine kinase domain, it was difficult to develop inhibitors of PTK7. To block the function of catalytically defective RPTKs, neutralizing Abs against these RPTKs have been developed and their value has been recognized in tumor therapy. For example, seribantumab (MM-121), a neutralizing mAb against ErbB3, significantly reduced tumor growth in xenograft models of lung and ovarian cancer derived from patients with carcinogenic neuregulin 1 (NRG1) fusion [22]. In addition, co-treatment with seribantumab and EGFR-neutralizing antibodies in EGFR mutant-bearing lung cancer cell lines and xenograft models, which became resistant to EGFR inhibitor/neutralizing antibodies, was able to overcome resistance to EGFR-targeted therapy and inhibit tumor growth [6]. As another example, a neutralizing mAb against EphA10 showed anti-tumor activity in a mouse model of TNBC, where EphA10 overexpression is known to act as a malignant factor [23]. In this regard, we developed mAbs that recognize the extracellular domain of PTK7 and analyzed their anti-tumor effects.

KYSE-30 cells do not express PTK7 at levels higher than that detected in other ESCC cells [12]. Thus, examining the effect of PTK7 loss-of-function on tumorigenesis in KYSE-30 cells is not optimal. Nevertheless, KYSE-30 cells have the advantage of being able to generate xenograft tumors in nude mice [24,25], and we found that PTK7 knockdown in KYSE-30 cells reduced tumorigenicity at the cellular level in xenograft mouse models [12]. Therefore, in this study, we used KYSE-30 cells to analyze the effect of PTK7 mAbs on the inhibition of tumorigenesis in ESCC.

We previously observed a decrease in cell growth after PTK7 knockdown in KYSE-30 cells grown in 2D cultures [12]. However, cells grown in 2D cultures are exposed to a different environment than the cells in tissues [26,27]. Hence, 3D models that can mimic in vivo conditions may be more beneficial for drug evaluation. Therefore, we evaluated the changes in the proliferation of KYSE-30 cells due to PTK7 mAbs in a 3D model using Matrigel, where, in addition to uncontrolled proliferation, cancer cells migrate into the extracellular matrix by adhesion of cell-surface adhesion receptors such as integrins [28]. We and others have shown that PTK7 is involved in the promotion of adhesion, wound healing, and migration in various cancers including ESCC [3,12,13,29,30,31]. The selected PTK7 mAbs significantly reduced the proliferation of KYSE-30 cells in 3D Matrigel, adhesion, wound healing, and migration of KYSE-30 cells, suggesting that PTK7 mAbs are able to block the oncogenic function of PTK7.

Metastasis, the leading cause of cancer death, occurs when cancer cells move through the blood to other areas via adhesion, migration, and invasion [32,33]. In the process of cancer metastasis, gelatinolytic MMPs degrade the extracellular matrix components that make up the basal lamina, particularly type IV collagen [34]. In addition, we previously found that PTK7 knockdown reduced the invasiveness of ESCC cells, and reduced MMP-9 expression plays an important role in reducing invasiveness [11]. In this study, we found that PTK7 mAbs significantly reduced invasion and MMP-9 secretion in KYSE-30 cells. Therefore, PTK7 mAbs were expected to inhibit invasion, at least in part, by reducing MMP-9 secretion.

In the process of cell migration and invasion, remodeling of the actin cytoskeleton occurs, particularly at the leading edges. In addition, actin filaments provide forces for cell movement through interactions with the ATPase myosin [35]. In this study, we confirmed that PTK7 mAb treatment impaired actin polymerization in the cortical regions of KYSE-30 cells as strongly as PTK7 knockdown did. Therefore, PTK7 mAbs are expected to reduce migration and invasion, in part by interfering with actin polymerization.

The ERK/MAPK signaling pathway is most commonly activated in cancer, affecting a wide range of processes, including cell proliferation, invasion, metastasis, and angiogenesis [36]. SRC, which is involved in cell proliferation, migration, and angiogenesis, is overexpressed in ESCC cells and tissues [37]. FAK, which is mainly involved in cell migration and invasion, is often activated and/or overexpressed in many ESCC cell lines and tissues, leading to metastasis [38]. In our previous studies, we showed that the phosphorylation of ERK, SRC, and FAK was inhibited by PTK7 knockdown in ESCC cells [3,11,12,39]. As expected, PTK7 mAbs reduced the phosphorylation of ERK, SRC, and FAK in KYSE-30 cells. Taken together, we confirmed that PTK7 mAbs, in the absence of a conjugated drug, can neutralize PTK7-mediated oncogenic functions and signaling pathways in ESCC cells.

In a mouse xenograft study using KYSE-30 cells, we observed that the PTK7 mAb-treated group had significantly reduced tumor volume and weight compared with that of the vehicle group. Additionally, IHC staining showed that Ki-67 expression was significantly reduced by PTK7 mAb treatment. These results indicate that PTK7 mAbs inhibit tumorigenesis not only at the cellular level but also in vivo.

One week after intraperitoneal injection of PTK7 mAbs twice per week for three weeks, xenograft tumors were significantly reduced compared to those in the vehicle group. However, our original plan was to sacrifice the mice two weeks after antibody injection. Unfortunately, inflammation and necrosis occurred in xenograft tumor tissues, especially in the control group, which showed rapid tumor growth. Since liquefaction and necrosis of tumor tissues in xenografts using KYSE-30 cells have been reported [40], this phenomenon, found in overgrown xenograft tumors, seems to be characteristic of KYSE-30 cells. Had there been no inflammation or necrosis of the xenograft tumor, the tumor could have grown for another week, and the difference in tumor growth between the control group and the PTK7 mAb-treated group would have been more significant.

Both PTK7 mAb-32 and PTK7 mAb-43 showed statistically significant anti-tumor activity at the cellular level and in vivo compared to the control. Although PTK7 mAb-32 and PTK7 mAb-43 showed no significant differences in some assays, PTK7 mAb-43 was more potent than PTK7 mAb-32 in invasion of KYSE-30 cells and Ki-67 expression levels in xenograft tumors. Therefore, it is expected that PTK7 mAb-43 may have better anti-tumor activity than PTK7 mAb-32.

We have shown that PTK7 enhances the activation of FGFR1 in both ligand-independent and ligand-dependent manners in ESCC cell lines [39]. It has recently been reported that PTK7 is involved in the activation of EGFR and AKT signaling in TNBC cell lines [13]. Thus, PTK7 is expected to play a role in regulating the signaling pathway through interaction with other RPTKs, such as FGFR1 and EGFR, or with other proteins. In addition, functional blocking of PTK7 by PTK7 knockdown or treatment of sPTK7 or PTK7-neutralizing Abs inhibits the activation of kinase insert domain receptor (KDR) by VEGF, reduces VEGF-induced cell migration and capillary-like tube formation in HUVEC cells, and inhibits angiogenesis in vivo [41,42]. Due to the mechanism of action of PTK7, counteracting PTK7 functions through PTK7 knockdown or treatment with PTK7 neutralizing antibody can significantly reduce tumorigenicity, though not completely regress the tumor cells. Therefore, we believe that PTK7 mAbs are useful in combination therapy with chemotherapeutic drugs and therapeutic antibodies.

When PTK7 mAbs were treated with KYSE-30 cells for cell signaling for 24 h, a decrease in PTK7 levels was consistently observed after treatment with either antibody, though PTK7 mAb-43 yielded better results than did PTK7 mAb-32. Binding of the PTK7 mAb to PTK7 is thus expected to induce internalization of the PTK7–PTK7 mAb complex. Considering that internalization induced by an antibody is an essential property for antibody-derived therapeutic agents such as ADC [43], PTK7 mAbs, particularly PTK7 mAb-43, hold great potential in ADC development.

## 4. Materials and Methods

### 4.1. Cell Culture

KYSE-30 cells were grown in DMEM/F12 medium (Gibco, Grand Island, NY, USA) supplemented with 2.5 mM sodium glutamine and 2% FBS (Gibco). Human embryonic kidney 293 (HEK293) cells expressing the SV40 T antigen (HEK293T) were grown in DMEM (Hyclone, South Logan, UT, USA) supplemented with 10% bovine serum (BS; Gibco). All media were supplemented with 100 U/mL penicillin and 100 μg/mL streptomycin and all cells were maintained at 37 °C in 5% CO_2_.

### 4.2. Generation of PTK7 Knockdown Lentiviruses and Infection of KYSE-30 Cells

The production of lentiviruses harboring PTK7 knockdown pLKO.1-shRNA-PTK7-6434 and pLKO.1-control vectors and the subsequent infection of ESCC cells was performed as previously described [12]. KYSE-30 cells infected with lentivirus were incubated with 1 μg/mL puromycin for 14 days, and puromycin-resistant colonies were pooled in a mixed culture.

### 4.3. Antibodies

Anti-phospho-p44/42 MAPK (ERK1/2) (Thr202/Tyr204) (9101s), anti-phospho-SRC (Y416) (2101s), anti-SRC (2109s), and anti-Ki-67 (9449s) antibodies was purchased from Cell Signaling Technology (Danvers, MA, USA); anti-ERK2 (BSM-52068R) antibody from Bioss (Boston, MA, USA); anti-phospho-FAK (Tyr397) (abt135) antibodies from Merck Millipore (Burlington, MA, USA); anti-FAK (sc-557) antibody from Santa Cruz Biotechnology (Santa Cruz, CA, USA); anti-GAPDH (abc2003) antibody from AbClone (Seoul, Korea); horseradish peroxidase (HRP)-conjugated goat anti-mouse IgG (K0211589) and rabbit IgG (K0211708) from KOMA Biotech (Seoul, Korea); and goat anti-mouse IgG (H + L) secondary antibody, HRP (nci1430kr) and goat anti-rabbit IgG (H + L) secondary antibody, and HRP (nci1460kr) from Invitrogen (Carlsbad, CA, USA). The generation of the anti-PTK7 antiserum has been described previously [44].

### 4.4. Production and Purification of Anti-PTK7 mAbs

Using purified human sPTK7 as an antigen, mice were immunized and hybridoma cell lines were produced using hybridoma technology by AbFrontier (Seoul, Korea). Hybridoma clones secreting mAbs with excellent binding affinity to sPTK7 were selected by ELISA. Small amounts of mAbs were purified from the serum-free conditioned medium of hybridoma cells incubated for 48 h. Proteins in the conditioned medium supplemented with 1 mM phenylmethanesulfonyl fluoride and 1 mM ethylenediaminetetraacetic acid (EDTA) were precipitated using 50% ammonium sulfate. The pellet was dissolved in 100 mM Tris-HCl, pH 8.0. Proteins were loaded onto a protein A/G agarose column. After washing with 100 mM Tris-HCl, pH 8.0, the mAb was eluted with 100 mM glycine, pH 3.0, neutralized with 1/10 volume of 1 M Tris-HCl, pH 8.0, and dialyzed with phosphate-buffered saline (PBS; 137 mM NaCl, 2.7 mM KCl, 10 mM Na_2_HPO_4_, and 1.8 mM KH_2_PO_4_). Large amounts of mAb were purified from ascites following intraperitoneal injection of hybridoma cells (Abclone, Seoul, Korea).

### 4.5. Binding Affinity Analysis of PTK7 mAbs Using SPR Method

Binding of PTK7 mAbs to His-tagged sPTK7 polypeptide was assayed using a Biacore T200 instrument (GE Healthcare, Chicago, IL, USA). Binding constant (*K*_D_) was measured at 25 °C with a CM5 chip, which was amino-coupled using a His Capture Kit. His-tagged sPTK7 polypeptide was injected for 30 s at a flow rate of 5 µL/min into the active flow cell. For kinetic analysis, PTK7 mAbs were serially diluted two-fold to 1.95–31.25 nM. HBS-EP+ buffer (10 mM HEPES, pH 7.4, 150 mM NaCl, 3 mM EDTA, and 0.05% (v/v) surfactant P20) was used as the running buffer. Surface regeneration was accomplished using 10 mM glycine-HCl, pH 1.5, after each cycle. Binding constants were determined using the BIA Evaluation software (version 1.0; GE Healthcare) and calculated using a 1:1 (Langmuir) binding fit model.

### 4.6. Cell Proliferation Assay in 3D Matrigel Culture

Cells were diluted to 6 × 10^4^ cells/mL with a 1:3 mixture of Matrigel (Corning, Bedford, MA, USA) and DMEM/F12 medium supplemented with 2% FBS. The cell–Matrigel mixture (50 μL) was plated in a 96-well flat-bottom plate (SPL, Pocheon, Korea) and incubated at 37 °C until solidification. Next, 50 μL of 2% FBS DMEM/F12 medium containing PTK7 mAbs (20 μg/mL) was added to the gel and incubated at 37 °C for 3 days. Live cells in 96 wells were stained by adding 10 μL WST-8 solution (5 mM WST-8 (Santa Cruz) and 200 μM 1-methoxy PMS (Selleckchem, Houston, TX, USA)) and incubating for 2 h. Absorbance was measured at 450 nm using a SpectraMax M3 microplate reader (Molecular Devices, San Jose, CA, USA).

### 4.7. Cell Adhesion Assay

Cell adhesion was performed as previously described with minor modifications [12]. Briefly, the cells were starved in serum-free DMEM/F12 medium for 24 h. Detached cells in serum-free DMEM/F12 medium (3 × 10^4^ cells/0.1 mL) were preincubated with PTK7 mAbs (10 μg/mL) for 30 min at 25 °C and plated onto rat-tail type I collagen-coated (1 μg/well) 96-well plates in the presence of 1% FBS for 1 h. The wells were washed thrice with PBS. Cells were fixed with 3.7% PFA in PBS and stained with 0.005% crystal violet. The stained cells were lysed with 1% sodium dodecyl sulfate (SDS), and the absorbance was measured at 600 nm.

### 4.8. Wound-Healing Assay

Cells were plated in 12-well dishes and grown to 70% confluence. The cells were starved for 24 h in serum-free DMEM/F12. Wounds were introduced by scraping the monolayer with a 1000p tip. Cells were incubated in 2% FBS DMEM/F12 medium containing PTK7 mAbs (10 μg/mL) for 48 h and imaged using light microscopy. The wound-healing area was evaluated using ImageJ software.

### 4.9. Chemotactic Migration and Invasion Assays

Chemotactic migration and invasion assays were performed using a Transwell system (Corning), as previously described, with minor modifications [12]. For the migration assay, the bottom surface of the Transwell plate was coated with 10 µL of 0.1% gelatin. The cells were starved in serum-free DMEM/F12 medium for 24 h. Detached cells in serum-free DMEM/F12 medium (7 × 10^4^/0.1 mL) were preincubated with PTK7 mAbs (10 μg/mL) for 30 min and loaded into the upper chamber of the Transwell. The lower compartment of each well was filled with 0.65 mL DMEM/F12 medium with 10% FBS as a chemoattractant. The chamber was incubated at 37 °C for 24 h. For the invasion assay, the upper surface of the Transwell plate was coated with 25 μg Matrigel in addition to the gelatin coating on its bottom surface. Invasion was performed using 2 × 10^5^ cells/0.1 mL and 48 h incubation, and the other procedures were the same as that of the migration assay. After incubation, the cells remaining in the upper compartment were removed using a cotton swab. Cells that migrated or invaded the bottom surface of the wells were fixed with 3.7% PFA in PBS and stained with 0.2% crystal violet. The stained cells were photographed and solubilized with 1% SDS, followed by measurement of absorbance at 600 nm.

### 4.10. Gelatin Zymography

Gelatin zymography was performed as previously described [45]. KYSE-30 cells were grown to 70% confluency in 12-well plates. The cells were washed thrice with PBS and incubated for 24 h in serum-free DMEM/F12 medium containing TNF-α (1 ng/mL) and PTK7 mAbs (10 μg/mL). The conditioned media were collected by centrifugation at 3000× *g* for 10 min and centrifuged again at 13,000× *g* for 10 min to remove cell debris. The conditioned media were loaded onto 9% SDS polyacrylamide gels containing 0.1% gelatin. Gels were incubated for 30 min at 25 °C in refolding buffer (50 mM Tris-HCl, pH 7.4, 100 mM NaCl, and 2.5% Triton X-100) twice and for 24 h at 37 °C in a reaction buffer (50 mM Tris-HCl, pH 7.4, and 10 mM CaCl_2_). After the reaction, the gel was stained with Coomassie brilliant blue. Band intensity in zymograms was measured using the ImageJ software.

### 4.11. Analysis of Actin Filament Distribution in Cells

Cells plated on a confocal dish (Corning) were incubated for 24 h in 2% FBS DMEM/F12 medium containing PTK7 mAbs (10 μg/mL). The cells were fixed with 3.7% PFA for 15 min and incubated at 25 °C for 1 h with 1 μg/mL of phalloidin-FITC (Thermo Fisher Scientific, Waltham, MA, USA) for actin filament staining and with 2.5 μg/mL of DAPI (Invitrogen) for nuclear staining. After staining, the cells were washed thrice with PBS and mounted with VECTASHIELD^®^ Antifade Mounting Medium (Vector Laboratories, Burlingame, CA, USA). Images were obtained using a confocal microscope (LSM700; Carl Zeiss, Feldbach, Switzerland) with 63X Plan-Apochromat objective lenses and Zen software (version 3.491; Carl Zeiss).

### 4.12. Western Blot Analysis

Cells were lysed with radioimmunoprecipitation assay buffer (50 mM Tris-HCl, pH 7.4, 150 mM NaCl, 1% NP-40, 0.5% sodium deoxycholate, and 0.1% SDS) containing 1 mM NaF and 1 mM NaVO_4_ for 15 min at 4 °C. Proteins in the cell lysates were resolved by SDS-PAGE and transferred to a PVDF membrane (Millipore). The blots were blocked with 5% skim milk and incubated with primary and secondary antibodies. Immunoreactive bands were detected using the West-Q PICO Dura ECL solution (GenDepot, Barker, TX, USA), Immobilon Western Chemiluminescent HRP Substrate (Millipore), and Amersham ImageQuant 800 (Cytiva, Marlborough, MA, USA). Protein bands obtained by Western blot were quantified using ImageJ software.

### 4.13. Analysis of Tumor Growth in Xenograft Mouse Model of ESCC

Four- to five-week-old male immune-deficient athymic nude (BALB/c nu/nu) mice were purchased from Orient Bio, Inc. (Gyeonggi, Korea). KYSE-30 cells (1 × 10^6^) were resuspended in 200 µL of a 1:1 mixture of Matrigel and PBS. The cell mixture was injected subcutaneously into the backs of mice. When tumor growth was evident, mice in the PTK7 mAb treatment group were intraperitoneally injected with 10 mg mAb/kg twice a week for three weeks, and mice in the control group were injected with the same volume of PBS. Tumor growth in each group was assessed by measuring tumor size twice per week using calipers (length/2 × width × width). The mice were sacrificed 1 week after the end of antibody treatment. Xenograft tumors were recovered to measure tumor weight, fixed in 4% PFA, and embedded in paraffin for histological and IHC analyses.

### 4.14. IHC Staining

Tissues in the paraffin blocks were cut to a thickness of 4 μm and subjected to deparaffinization with xylene and dehydration with ethanol. For antigen retrieval, tissue sections were boiled in sodium citrate buffer (10 mM sodium citrate and 0.05% Tween 20, pH 6.0) for 10 min on a hot plate and then incubated at 25 °C for 20 min. After washing with PBS, the tissue sections were quenched in 3% H_2_O_2_ in PBS and incubated in blocking buffer (PBS, 1% BSA, and 0.5% Triton X-100) at 25 °C for 30 min. After washing with PBS, the tissue sections were incubated overnight with the primary antibody in a humid chamber at 4 °C. Tissue sections were washed with PBS and incubated with an HRP-conjugated secondary antibody (Invitrogen) in a humid chamber at 25 °C for 1 h. Tissue sections were washed with PBS, stained with 3,3-diaminobenzidine chromogen (Thermo) for 5 min at 25 °C, washed with distilled water, and counterstained with hematoxylin (DAKO, Carpinteria, CA, USA). Stained tissue sections were mounted using Permount (Thermo Fisher Scientific), and the stained images were examined by light microscopy. IHC staining was performed using the IHC profiler plugin of the ImageJ software [46].

### 4.15. Ethics Statement

The protocol for the animal studies was approved by the Institutional Animal Care and Use Committee of Yonsei University (approval number: IACUC-A-202104-1244-05). Animal studies were performed in accordance with the committee guidelines.

### 4.16. Statistical Analysis

All statistical analyses were performed using Microsoft Excel (Microsoft Corp., Redmond, WA, USA). Statistical significance was analyzed using Student’s *t*-test for homoscedastic variance and Welch’s *t*-test for heteroscedastic variance. Homoscedasticity or heteroscedasticity of variance was determined by two-tailed F-test. For all tests, *p*-values less than 0.05 were considered statistically significant.

## 5. Conclusions

We previously found that knockdown of PTK7 reduces the oncogenic phenotypes of ESCC cells and inhibits tumor growth in xenograft mice with KYSE-30 cells. In this study, we showed that mAbs against the extracellular domain of PTK7 significantly reduced the proliferation, adhesion, wound healing, migration, and invasion of ESCC KYSE-30 cells. PTK7 mAbs decreased MMP-9 secretion, which is essential for the degradation of the basal lamina, and reduced actin polymerization, which is important for cell locomotion. Furthermore, PTK7 mAbs significantly reduced tumor growth and Ki-67 expression levels in xenograft tumor mice of KYSE-30 cells. Our results demonstrated that PTK7 mAbs potently neutralized the oncogenic functions of PTK7 at the cellular level and in an in vivo mouse model. Therefore, we propose that PTK7 mAbs can be used as lead molecules to develop humanized PTK7 antibodies as novel therapeutics for PTK7-positive cancers, including ESCC.

## Data Availability

Data are contained within the article.

## Supplementary Materials

The following are available online at https://www.mdpi.com/article/10.3390/ijms232012195/s1.
