# Supplementary material for: Anti-PTK7 Monoclonal Antibodies Exhibit Anti-Tumor Activity at the Cellular Level and in Mouse Xenograft Models of Esophageal Squamous Cell Carcinoma
Source: Int J Mol Sci. 2022 Oct 13;23(20):12195. doi: 10.3390/ijms232012195 (PMC9603586; doi:10.3390/ijms232012195)
Supplement: Supplementary file 1 [file ijms-23-12195-s001.zip › ijms-1899014-supplementary.pdf]

Supplementary Materials

Anti-PTK7 monoclonal antibodies exhibit anti-tumor activity at the cellular level and in mouse xenograft models of esophageal squamous cell carcinoma

Jae Hoon Kim[1], Won-Sik Shin[1], Se-Ra Lee[2], Sanggil Kim[2], So-Young Choi[2] and Seung-Teak Lee[1,*]

1 Department of Biochemistry, College of Life Science and Biotechnology, Yonsei University, Seoul 03722, Republic of Korea
2 New Drug Development Center, Osong Medical Innovation Foundation, Cheongju, Chungbuk 28160, Republic of Korea
* Correspondence: stlee@yonsei.ac.kr; Tel.: +82-221232703

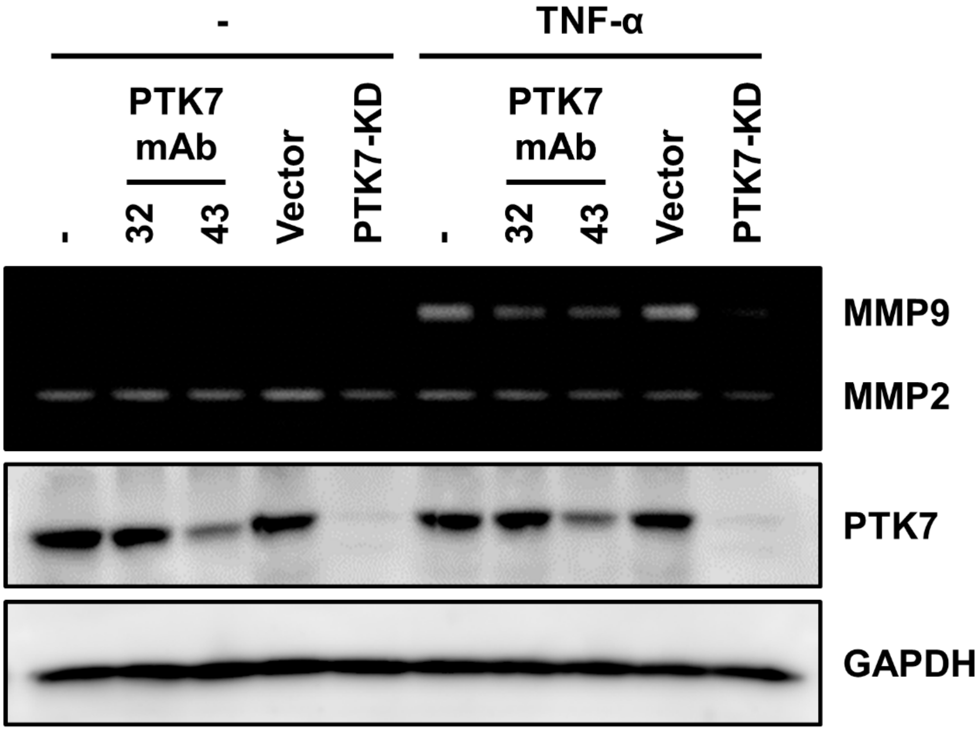

Supplementary Figure S1. Secretion of matrix metalloproteinase (MMP)-9 is enhanced by TNF-α treatment in KYSE-30 cells. MMP-2 and MMP-9 levels in the conditioned medium of KYSE-30 cells, which were incubated in serum-free DMEM/F12 medium with or without PTK7 monoclonal antibodies (10 μg/mL) in the absence or the presence of TNF-α (1 ng/mL) for 24 h, were analyzed using gelatin zymography.
